# Supplementary material for: Cryptococcus neoformans releases proteins during intracellular residence that affect the outcome of the fungal–macrophage interaction
Source: Microlife. 2022 Sep 21;3:uqac015. doi: 10.1093/femsml/uqac015 (PMC9552768; doi:10.1093/femsml/uqac015)
Supplement: uqac015_Supplemental_Files [file uqac015_supplemental_files.zip › SFig6. Bioinformatics-supplementary data-compre.pdf]

Dataset	% Discovered	Upregulated	Downregulated
31860441 (CSF)	100	77	149
31860441 (Macrophage)	75.22	39	131
33688010 (Temp)	87.17	54	143
28376087 (Temp)	61.06	67	71
27094327 (Temp)	48.23	31	78
31666517 (Temp)	53.98	53	69

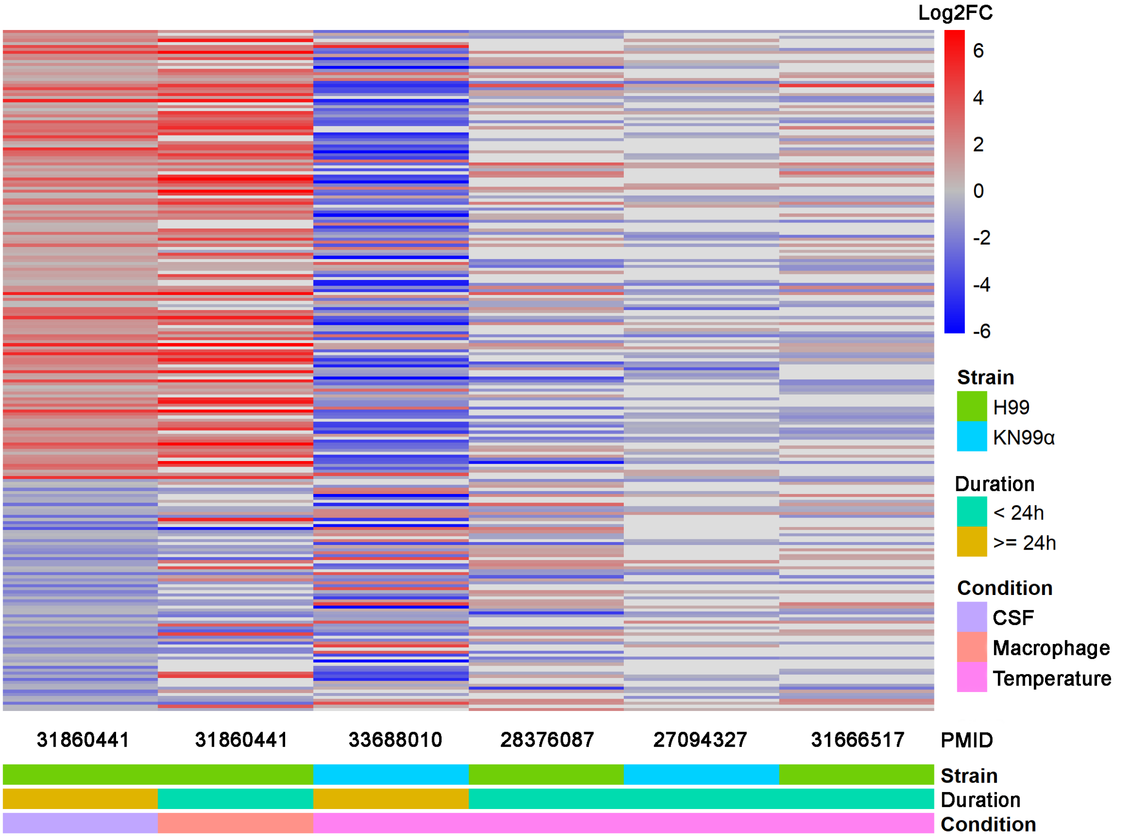

Reference	Strain	Conditions	Duration	Log_2FC
31860441	H99	Rabbit CSF *in vivo*	24 h	0.24
33688010	KN99α	37 °C|5% CO_2|DMEM	24 h	2.32
27094327	KN99α	37 °C|5% CO_2|DMEM	1.5, 3, 8, 24 h	0.33
