# Supplementary figures and images for: Cryptococcus neoformans releases proteins during intracellular residence that affect the outcome of the fungal–macrophage interaction
Source: Microlife. 2022 Sep 21;3:uqac015. doi: 10.1093/femsml/uqac015 (PMC9552768; doi:10.1093/femsml/uqac015)

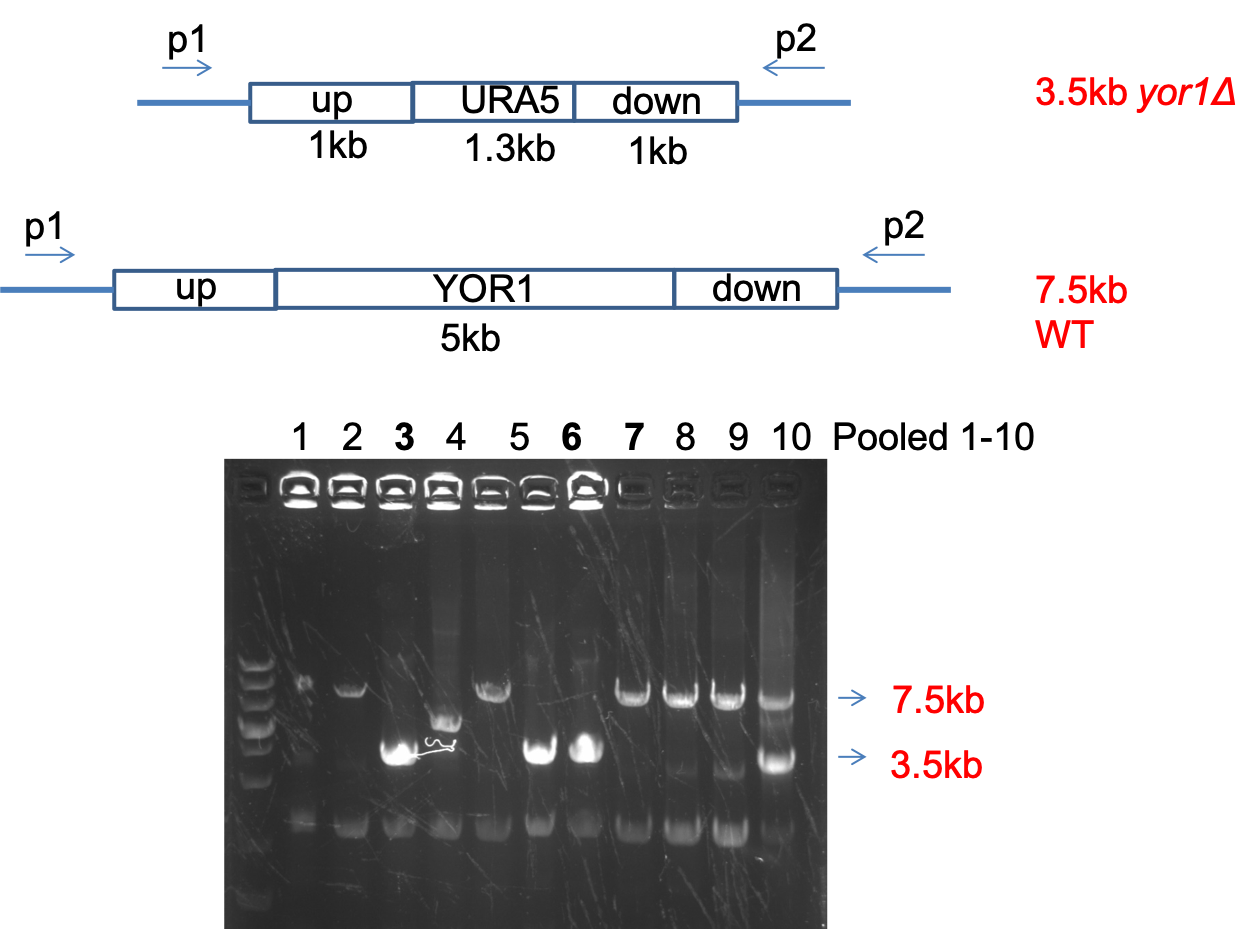

Supplement: uqac015_Supplemental_Files [file uqac015_supplemental_files.zip › SFig1-supplementary data.png]

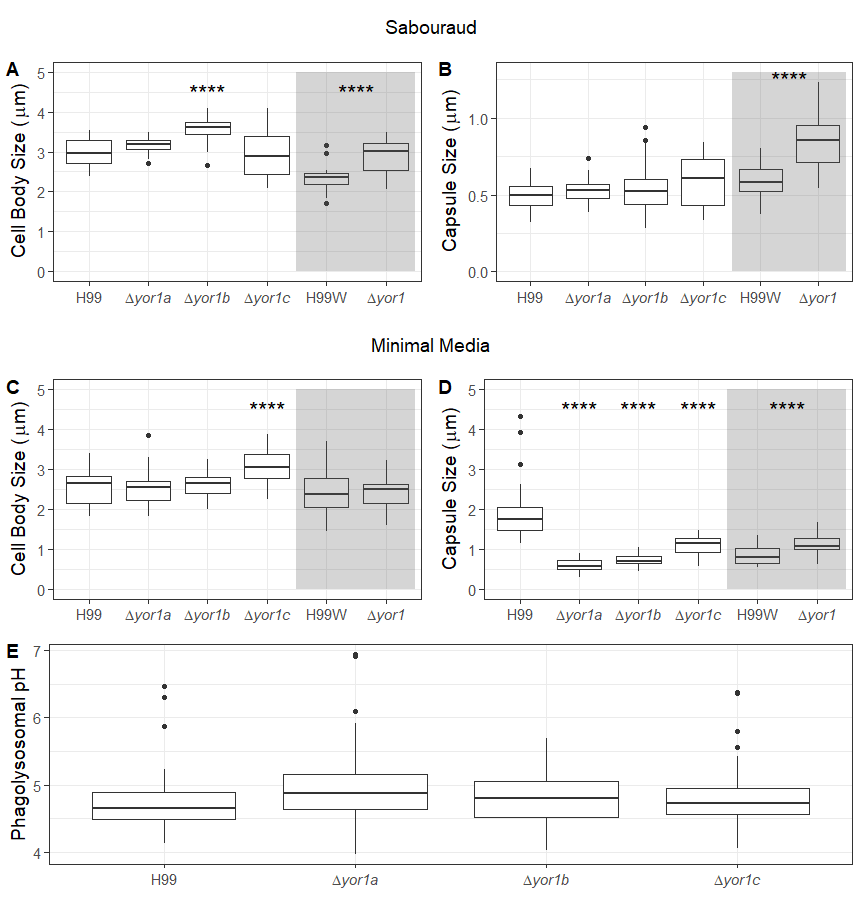

Supplement: uqac015_Supplemental_Files [file uqac015_supplemental_files.zip › SFig2-supplementary data.tiff]

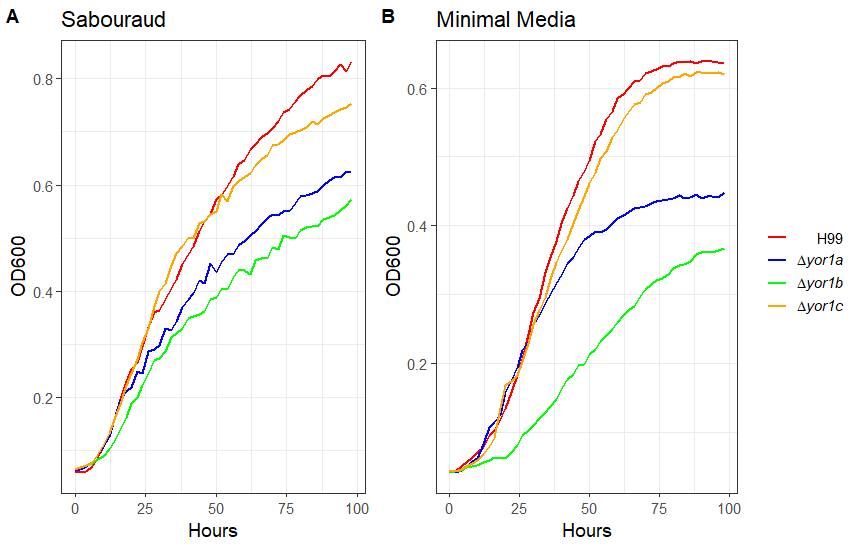

Supplement: uqac015_Supplemental_Files [file uqac015_supplemental_files.zip › SFig3-supplementary data.tiff]

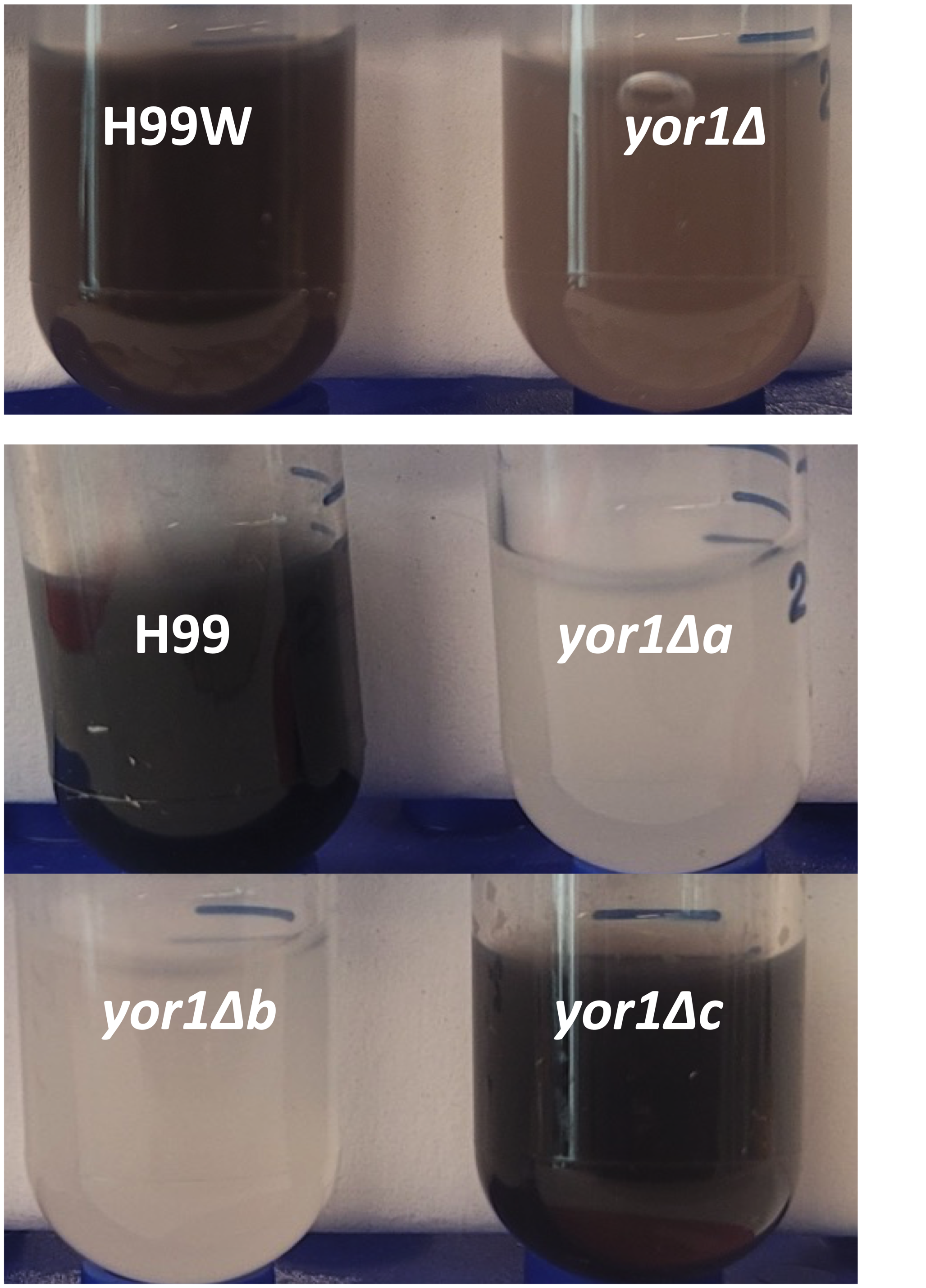

Supplement: uqac015_Supplemental_Files [file uqac015_supplemental_files.zip › SFig4. Melanization panel-supplementary data.tiff]

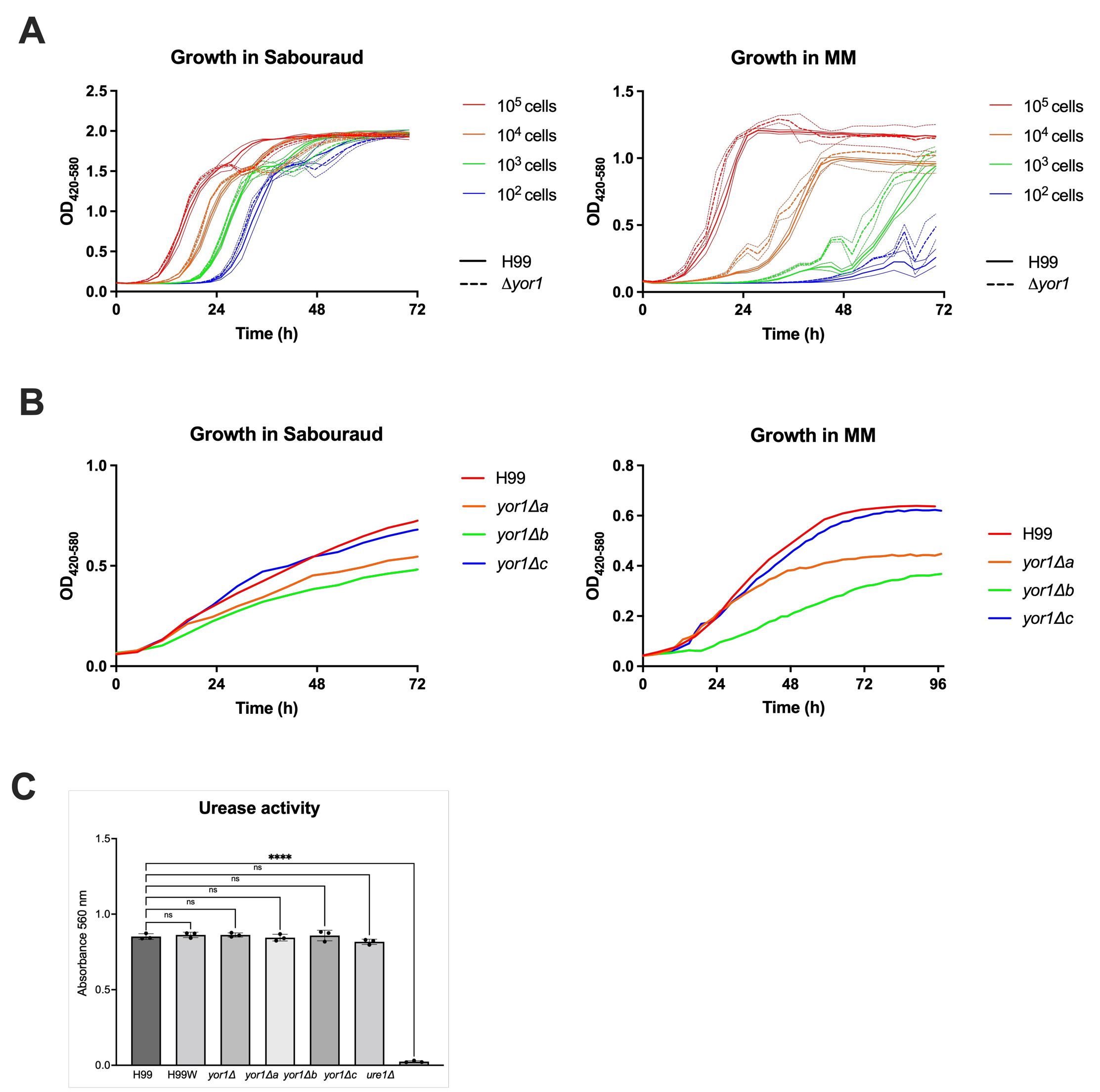

Supplement: uqac015_Supplemental_Files [file uqac015_supplemental_files.zip › SFig5-Apr2022-supplementary data.tiff]
